# Supplementary material for: Mosaic evolution in an asymmetrically feathered troodontid dinosaur with transitional features
Source: Nat Commun. 2017 May 2;8:14972. doi: 10.1038/ncomms14972 (PMC5418581; doi:10.1038/ncomms14972)
Supplement: Supplementary Information — Supplementary Figure, Supplementary Tables, Supplementary Note and Supplementary References [file ncomms14972-s1.pdf]

Supplementary Note 1

Additional osteological description

The text below provides additional details of *Jianianhualong* that were not pertinent to the salient osteological description provided in the main text.

Cranium

The anterodorsal margin of the rostral ramus of the maxilla is straight and is continuous with the dorsal margin of the maxillary ascending process in lateral view; in other troodontids including *Sinovenator*[1], the dorsal margin is concave. The lateral surface of the rostral ramus is depressed, but this might be a preservational artefact. The ascending process has an extremely narrow lateral lamina and a broad medial lamina. The posterior end of the medial lamina is bifid for receiving the anterior process of the lacrimal. The jugal ramus of the maxilla has almost parallel dorsal and ventral edges along most of its length; posteriorly it retains the same depth, unlike the dorsoventrally narrow and transversely thick jugal ramus in *Sinovenator*[2,3]. However, a posteriorly deep jugal ramus appears to characterize derived troodontids[1]. As in derived troodontids such as *Zanabazar junior*[1,4], the anterior portion of the jugal ramus is relatively deep dorsoventrally compared to the depth of the antorbital fossa above, whereas it is shallow in *Sinovenator*[2]. There is a ridge that rises across the mid-line of the jugal ramus and beneath it are at least two elongate foramina – this ridge is absent in *Byronosaurus*[5], *Sinovenator*[3], *Sinusonasus*[6], *Xixiasaurus*[7], and *Zanabazar*[4], but a similar weak ridge is present in *Gobivenator*[8]. The posterior end of the jugal ramus is trifid and the dorsal process is the longest.

The antorbital fossa has a sharp rim as in *Byronosaurus*[9,10], some dromaeosaurids[11], oviraptorosaurians and therizinosauroids[12]. The antorbital fenestra occupies more than half of the antorbital fossa as in *Sinovenator*[2], unlike the proportionally smaller one in *Byronosaurus*[10]. There is a relatively narrow interfenestral bar, although it is not as narrow as in *Sinovenator*[2], and it is inset medially from the lateral surface of the maxilla.

The long anterior process of the lacrimal has a relatively wide medial lamina contributing to the dorsal portion of the antorbital fossa, and the medial lamina covers most of the length of the anterior process except the most anterior portion. The posterior process is less than half the length of the anterior process, and its posterior end is bifid, with a shorter ventral ramus. The ventral end of the descending process is blunt and has a ventroposteriorly-directed concave margin.

The anterior margin of the frontal is oblique, with the medial end extending more anteriorly than the lateral end. The narrowest region of the frontal is at the level of the orbital mid-length. The posterior margin of the frontal is convex in dorsal or ventral view. The internal surface of the frontal has a prominent crista cranium along the orbital edge. The anterior end of the crista cranium is confluent with the anterolateral end of the frontal, and diverges from the orbital edge posteriorly until it is ~5 mm away from the orbital edge at its posterior end. The crista cranium marks the anteriormost portion of the lateral margin of the frontal, and has a relatively simple frontal-lacrimal contact rather than the complex one seen in *Troodon*[13]. The internal surface of the frontal provides some information on the braincase: the olfactory tract is wide transversely and the cerebral hemisphere is enlarged transversely, particularly posteriorly.

The anterior process of the left jugal curves gently dorsally; its tapered end fits into the dorsal notch of the posterior end of the maxillary jugal ramus. The ascending process extends

posterodorsally to form a smooth transition from the orbital rim, and its distal portion is sub-triangular in cross section. The jugal expands slightly ventrally and laterally to form a small cornual process at the junction area of the anterior and ascending processes.

The angle between the frontal and jugal processes of the left postorbital is ~145 degrees, indicating that the frontal process is upturned.

Both pterygoids are preserved, but neither is well exposed. The quadrate ramus of the pterygoid appears to be small, matching the equally small pterygoid ramus of the quadrate. The pterygoid shaft is sub-triangular in cross section and bears a shallow and long ventral flange.

The dentary is about the same length as the postdentary region measured along the dorsal margin. Both the dorsal and ventral margins of the dentary are nearly straight in lateral view as in unenlagiids. The anterior end of the dentary is not slightly downturned as in *Sinovenator*[2], but the dorsal margin of the anterior portion of the dentary is convex in lateral view. The dorsal border of the groove on the lateral surface of the dentary is narrow anteriorly is close to the dorsal margin of the dentary with a ridge bordering the groove for its posterior half. This groove extends from the anterior one-third of the dentary and terminates near the external mandibular fenestra. Most of the latter is floored by the prearticular. Many small, round vascular foramina are on the lateral surface of the dentary close to its anterior end; posteriorly they are larger in size and more elongate, and most are located within the groove as in other troodontids[10]. The posterior end of the dentary is about twice as deep as the anterior end, and it is highly angled with a long, pointed ventral process. On the medial surface of the dentary, a Meckelian canal extends anteriorly close to the mandibular symphysis. It is positioned close to the ventral margin of the dentary, is narrow and deep anteriorly, and becomes much wider posteriorly.

The anterior process of the surangular contacts the dentary anteriorly and the angular ventrally, and forms the dorsal and posterior border of the external mandibular fenestra. Two surangular foramina are present, the posterior one of which is larger in size. The dorsal ramus of the surangular is broad transversely, roofing the internal mandibular fossa.

The anterior process of the angular is robust and curves dorsally to form the anterior and ventral border of the large external mandibular fenestra. The posterior portion of the angular seems to be narrow and terminates well anterior to the posterior end of the mandible.

The anterior process of the splenial is shallow, but its anterior extent is not clear; the posterior process lacks a deep V-shaped notch as in many other theropods such as *Deinonychus*[14]. The posterior extremity is wider mediolaterally than dorsoventrally, with a flat dorsal surface for articulating with the ventral margin of the anterior portion of the angular.

The anterior and posterior portions of the prearticular are both dorsoventrally expanded relative to the middle of the bone. Anteriorly the prearticular contacts both the angular and splenial along a straight joint that is ventrodistally orientated in lateral view. Ventrally it contacts the angular along a curved joint that is ventrally convex in lateral view. Distally the prearticular contacts the articular along a straight joint that appears to be vertically orientated in lateral view.

There are some morphological variations along the maxillary tooth row. As in other troodontids[1], the anterior teeth are inferred (based on the tooth sockets) to be closely packed, whereas the middle and posterior teeth are more widely spaced. The most posterior teeth are considerably smaller in size than more anterior teeth. The middle and posterior teeth have proportionally shorter crowns than the anterior teeth. The slenderness index (SI: a ratio of maximum apicobasal length to maximum mesiodistal width of the crown) of the first and sixth preserved teeth from the anterior end are 1.5 and 1.2. In comparison, the SI of an anterior maxillary tooth in a *Microraptor* specimen (IVPP V13476) is

2.6. Short-crowned dentition characterizes the Troodontidae. The most anterior preserved maxillary tooth lacks serrations on both mesial and distal carinae, but the remaining teeth have serrations on the distal carina. The serrations are relatively fine as in *Sinovenator*[2], unlike the coarse ones in derived troodontids[1]. Similar to other Jehol troodontids [2], the posteriormost maxillary tooth is located close to the posterior end of the maxillary jugal ramus (thus close to the orbital bar), unlike most other coelurosaurians in which the maxillary tooth row terminates well anterior to the orbital bar. There are some features of the dentary teeth that are not seen in the maxillary teeth. The anteriormost dentary teeth are slightly procumbent, which is a feature that also appears to be present in some Jehol dromaeosaurid specimens[2]. The anterior dentary teeth are closely packed, whereas the middle and posterior ones have relatively wide inter-dental spaces, confirming the inferred distribution pattern for the maxillary teeth.

Postcranium

The third cervical is moderately elongate, and the centrum is 12 mm long. The associated cervical rib is preserved separately, and is long (covering both the third and fourth cervical vertebrae) and slender (with a small capitulum and tuberculum). The neural spine of the third cervical is relatively short anteroposteriorly. The fourth cervical is slightly longer than the third one and its associated cervical rib is similar to the third one, although it is more robust. The neural spine of the fourth cervical is rectangular in lateral view, with its anteroposterior length ~1.5 times its dorsoventral depth. The cervical ribs of the fifth and sixth cervicals are only slightly longer than their corresponding cervical centra. The most posterior cervicals are significantly shorter than the fifth and sixth cervical vertebrae. Their associated cervical ribs are about the same lengths as their corresponding centra, and each has an expanded capitulum and tuberculum.

The middle and posterior dorsal vertebrae each have a shallow centrum (the most posterior ones have a central length/height ratio of ~2.0, as measured from their posterior ends) as in other small-sized deinonychosaurs[2]. The dorsal vertebrae have fan-shaped neural spines, the posterior ones of which are located on the posterior halves of the corresponding centra, a feature also seen in other basal paravians[2]. The first pair are short, the second pair are twice the length of the first pair, the third and fourth pairs are the longest, and the more posterior ones are much shorter, with the shortest one measuring ~30% of the longest one.

The most anterior imbricated gastral basket elements are level with the anteriormost dorsal ribs and the posteriormost ones level with the pubes. As in other theropods including *Velociraptor*[15], the first pair of the gastral elements is much more robust than the other gastral elements, with their medial ends much thicker than their lateral ends.

The most significant change in caudal length occurs at the 9th caudal which is ~1.7 times the anteroposterior central length of the 8th caudal. The transition point is likely to be between the 9th and 10th caudal vertebra, as the former has a very small transverse process whilst the latter lacks one. The centra remain rectangular in transverse cross section until the 23rd caudal, whereas more posterior caudal centra are strongly compressed laterally so that their transverse cross sections are sub-triangular with narrow ventral surfaces. In *Sinovenator*, this strong lateral compression starts approximately two-thirds of the way down the caudal series. The centra of the 24th and subsequent caudals also differ from those of more anterior caudals in having their anterior articular ends distinctly deeper than their posterior ends in lateral view. The middle caudal centra have transversely flat ventral surfaces, although the presence of grooves along the ventral surfaces of more anterior caudal centra,

which would be expected in coelurosaurs, is not known.

As in other paravians, the caudal centra display a striking increase in anteroposterior length along the caudal series, with the 15th caudal centrum - which is among the longest caudals at 23.3 mm - approximately twice as long as the 6th caudal. As in other troodontids[1], the zygapophyses are small and nearly horizontal in orientation. In contrast, most other tetanurans have long prezygapophyses. Neural spines are present in the anterior eight caudals only, but it is not known whether a groove is present along the dorsal surface of the caudal neural arch, as in other troodontids[1]. All preserved chevrons are plate-like. The anteriormost chevrons are much longer dorsoventrally than anteroposteriorly. The chevrons associated with the sixth through ninth caudal vertebrae are longer anteroposteriorly than deep dorsoventrally, although they have considerable dorsoventral depth. The one between the ninth and tenth caudals has a very shallow dorsoventral depth relative to its anteroposterior length. More posterior chevrons are somewhat flattened dorsoventrally, with sub-equal anteroposteriorly elongate anterior and posterior processes. Although most chevrons are long anteroposteriorly, there are gaps between the chevrons, unlike in *Sinusonasus magnodens*[6] where the chevrons contact each other underneath the caudal vertebrae.

The ulna has a moderate olecranon process, distal to which seems to be a prominent anterior flange. The mid-shaft of the ulna is thin (4.6 mm in diameter), but thickens gradually towards both ends. The distal end of the ulna is transversely tapered and has a distally convex articular surface.

The distal half of the radius is somewhat compressed transversely and the flattened distal end has a convex distal articular surface in medial and lateral view.

The distal end of metacarpal II is strongly ginglymoid, with an oblique medial hemi-condyle, and a lateral hemi-condyle that extends more distally than the medial one.

Metacarpal III is nearly as robust as metacarpal II. In dorsal view, metacarpal III is widest transversely at the proximal end and narrows gradually distally, although the distal end is wider transversely than the shaft. There appears to be a longitudinal groove along the dorsal surface near the distal end.

There is a longitudinal groove along most of the dorsal surface of metacarpal IV. The distal end extends slightly beyond that of metacarpal III, a feature unknown in any non-avialan theropod, but present in enantiornithine birds[16].

The semilunate carpal is relatively small in size, and it contacts both the proximal ends of the metacarpals II and III. The proximal surface of the semilunate carpal is strongly convex transversely and bears a distinctive transverse groove. There is small carpal sitting at the proximal end of metacarpal IV, which we identify as distal carpal 4. A carpal intermediate in size is displaced to the lateral side of the right wrist, and is tentatively identified as the right radiale.

The proximal end of metacarpal IV is positioned slightly distal to that of metacarpal III as in many paravian theropods.

The distal articulation of manual phalanx II-1 has hemi-condyles with weak dorsal extensions but prominent ventral and proximal extensions. Both the medial and lateral collateral ligament pits are present and dorsally positioned. The medial one appears to be deeper unlike manual phalanx II-1 of most theropods but as in manual phalanx IV-1 of most theropods.

Manual phalanx IV-2 has a prominent proximoventral heel and a deep groove along the ventral surface of the distal half. The distal articulation of manual phalanx IV-3 has considerable dorsal and ventral expansions.

The second digit has the largest ungual, and ungual 4 the smallest. Comparatively, the tips of

unguals 2 and 3 extend more ventrally than ungual 4, which is slightly arched dorsally. Dorsally arched unguals are also known in some derived maniraptorans including the dromaeosaurids[17], but in the latter group, all manual unguals are strongly arched dorsally.

The ischial peduncle of the pubis is short anteroposteriorly, with a subrectangular articular facet that faces posteriorly. The ischial articulation is somewhat hooked, extending ventrally to form a pointed process just posterior to the proximal pubic shaft. There is a large depression on the lateral surface immediately anterior to the ischial articulation.

The proximal end of metatarsal II has a conspicuous medial expansion. In posteroventral view, the proximal half of metatarsal II is much thinner than those of metatarsals III and IV (a transverse width ratio of metatarsal II to III and IV about one third and less than one fourth, respectively). The distal half is thinner than the corresponding regions of metatarsals III and IV, but not to the degree of the proximal half. Metatarsal III is 72% of femoral length, proportionally shorter than that of *Sinovenator* in which this percentage is close to 90%. Its distal end lacks a ginglymoid articulation.

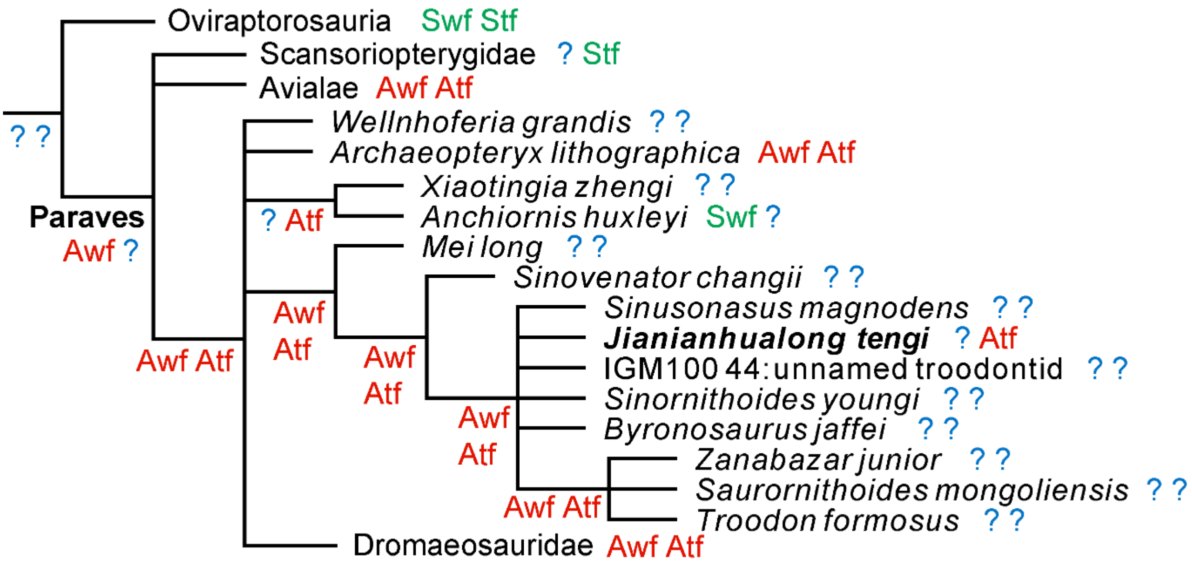

Supplementary Figure 1.

Parsimony-based ancestral state reconstruction of paravian arm and tail feather asymmetry.

Ancestral state reconstruction produced in the software *Mesquite* using the tree topology shown in Figure 6.

Supplementary Table 1. Selected measurements of *Jianianhualong tengi* holotype. All measurements are lengths in mm unless otherwise noted; * indicates estimated value.

Measurement	Value (left element/right element)
Maxilla	42.3/?
Jugal	38.5/?
Frontal	36.1
Mandible	87.0
Presacral vertebrae 2-6	54.8 (the sixth: 17; the seventh: 13)
Presacral vertebrae 7-12	12.2/12.2/10.7/10.7/10.1/9.4
Presacral vertebrae 13-16	53.7
Presacral vertebrae 17	12.8
Presacral vertebrae 18-19	26.8
Presacral vertebrae 20-22	14.2/14.0/15.3
Caudal ?6-28	7.4/7.4/7.4*/12.7/13.0/21.4/19.4/21.7/22.7/22.8/22.7/22.8/22.4/21/20.9/20*/20.5/20.3/19.3/18.2/16.5/15/1 = 406.7 + (30* + 25*) = 461.7 (total length)
Cervical ribs 1-6	19.5/?/>19.5/?/16.5/13.8
Dorsal ribs 1-9	30/60/78/85/55/50/45/35/25
Scapula	79.4/79.3
Humerus	?/80.8
Ulna	69/71.2
Radius	67.5/68
MC II	14.3/14.7
MC III	31.3/?
MC IV	30.6/?
Manual phalanx II-1	26.6/28
Manual phalanx II-2	19.7/22*
Manual phalanx III-1	23.3/23.3
Manual phalanx III-2	32.8/31.7
Manual phalanx III-3	23/23.5
Manual phalanx IV-1	8.2/?
Manual phalanx IV-2	7.4*/6.7
Manual phalanx IV-3	20.6/20.6
Manual phalanx IV-4	17/16.1
Ilium	?/75
Pubis	91*
Ischium	44.2/44
Femur	117/116
Metatarsal I	7.8/?
Metatarsal II	74.3/74.7
Metatarsal III	84.3/84.4
Metatarsal IV	80.9/79.1
Metatarsal V	33.8/34

Pedal phalanx I-1	14.6/13.4
Pedal phalanx I-2	10.5*/13
Pedal phalanx II-1	20.1
Pedal phalanx II-2	14
Pedal phalanx II-3	>28
Pedal phalanx IV-1	20

Supplementary Table 2. Phylogenetic data matrix of paravian arm and tail feather symmetry. The phylogenetic data matrix includes the arm and tail feather symmetry of the paravian taxa shown in Figure 6.

Taxon	Arm feather asymmetry: absent (0); present (1)	Tail feather asymmetry: absent (0); present (1)
Oviraptorosauria	0	0
Scansoriopterygidae	?	0
Avialae	1	1
Wellnhoferia grandis	?	?
Archaeopteryx lithographica	1	1
Xiaotingia zhengi	?	?
Anchiornis huxleyi	0	?
Mei long	?	?
Sinovenator changii	?	?
Sinusonasus magnodens	?	?
Jianianhualong tengi	?	1
IGM 100 44: unnamed troodontid	?	?
Sinornithoides youngi	?	?
Byronosaurus jaffei	?	?
Zanabazar junior	?	?
Saurornithoides mongoliensis	?	?
Troodon formosus	?	?
Dromaeosauridae	1	1

Supplementary References

1 Makovicky, P. J. & Norell, M. A. in *The Dinosauria 2nd edn* (eds D B Weishampel, P Dodson, & H Osmólska) 184-195 (University of California Press, 2004).

2 Xu, X. *Deinonychosaurian fossils from the Jehol Group of western Liaoning and the coelurosaurian evolution* Ph.D. Dissertation thesis, Chinese Academy of Sciences, (2002).

3 Xu, X., Norell, M. A., Wang, X. L., Makovicky, P. J. & Wu, X. C. A basal troodontid from the Early Cretaceous of China. *Nature* **415**, 780-784 (2002).

4 Norell, M. A. *et al.* A review of the Mongolian Cretaceous dinosaur *Saurornithoides* (Troodontidae: Theropoda). *American Museum Novitates* **3654**, 1-63 (2009).

5 Bever, G. & Norell, M. A. The perinate skull of *Byronosaurus* (Troodontidae) with observations on the cranial ontogeny of paravian theropods. *American Museum Novitates* **3657**, 1-51 (2009).

6 Xu, X. & Wang, X. L. A new troodontid (Theropoda: Troodontidae) from the Lower Cretaceous Yixian Formation of western Liaoning, China. *Acta Geologica Sinica (English Edition)* **78**, 22-26 (2004).

7 Lü, J. C. *et al.* A new troodontid (Theropoda: Troodontidae) from the Late Cretaceous of central China, and the radiation of Asian troodontids. *Acta Palaeontologica Polonica* **55**, 381-388 (2010).

8 Tsuihiji, T. *et al.* An exquisitely preserved troodontid theropod with new information on the palatal structure from the Upper Cretaceous of Mongolia. *Naturwissenschaften* **101**, 131-142 (2014).

9 Norell, M. A., Makovicky, P. J. & Clark, J. M. A new troodontid theropod from Ukhaa Tolgod, Mongolia. *Journal of Vertebrate Paleontology* **20**, 7-11 (2000).

10 Makovicky, P. J., Norell, M. A., Clark, J. M. & Rowe, T. Osteology and relationships of *Byronosaurus jaffei* (Theropda: Troodontidae). *American Museum Novitates* **3402**, 1-32 (2003).

11 Xu, X. & Wu, X. C. Cranial morphology of *Sinornithosaurus millenii* Xu et al. 1999 (Dinosauria, Theropoda, Dromaeosauridae) from the Yixian Formation of Liaoning, China. *Canadian Journal of Earth Sciences* **38**, 1739-1752 (2001).

12 Clark, J. M., Altangerel, P. & Norell, M. A. The skull of *Erlicosaurus andrewsi*, a Late Cretaceous "segnosaur" (Theropoda: Therizinosauridae) from Mongolia. *American Museum Novitates* **3115**, 1-39 (1994).

13 Currie, P. J. Cranial anatomy of *Stenonychosaurus inequalis* (Saurischia, Theropoda) and its bearing on the origin of birds. *Canadian Journal of Earth Sciences* **22**, 1643-1658 (1985).

14 Ostrom, J. H. Osteology of *Deinonychus antirrhopus*, an unusual theropod from the Lower Cretaceous of Montana. *Bulletin of the Peabody Museum of Natural History, Yale University* **30**, 1-165 (1969).

15 Norell, M. & Makovicky, P. J. Important features of the dromaeosaur skeleton: information from a new specimen. *American Museum Novitates* **3215**, 1-28 (1997).

16 Chiappe, L. M. Late Cretaceous birds of southern South America: anatomy and systematics of enantiornithes and *Patagopteryx deferrariisi*. *Munchner Geowiss, Abh.* **30**, 203-244 (1996).

17 Senter, P., Barsbold, R., Britt, B. & Burnham, D. A. Systematics and evolution of Dromaeosauridae (Dinosauria, theropoda). *Bulletin of the Gunma Museum of Natural History* **8**, 1-20 (2004).
